# Synthesis and Characterization of a Novel Concentration-Independent Fluorescent Chloride Indicator, ABP-Dextran, Optimized for Extracellular Chloride Measurement

**DOI:** 10.3390/biom14010077

**Published:** 2024-01-08

**Authors:** Kieran P. Normoyle, Kyle P. Lillis, Kevin J. Staley

**Affiliations:** 1 Massachusetts General Hospital, Department of Neurology, 55 Fruit Street, Boston, MA 02114, USA; kieran.normoyle@mgh.harvard.edu (K.P.N.); klillis@mgh.harvard.edu (K.P.L.); 2 Harvard Medical School, Department of Neurology, 77 Louis Pasteur Avenue, Boston, MA 02115, USA

**Keywords:** extracellular chloride, extracellular matrix, fluorescent chloride sensor, fluorescence lifetime imaging, fluorescent dextran

## Abstract

GABA, the primary inhibitory neurotransmitter, stimulates GABAA receptors (GABAARs) to increase the chloride conductance of the cytosolic membrane. The driving forces for membrane chloride currents are determined by the local differences between intracellular and extracellular chloride concentrations (Cl_i_ and Cl_o_, respectively). While several strategies exist for the measurement of Cl_i_, the field lacks tools for non-invasive measurement of Cl_o_. We present the design and development of a **f**luorescent **l**ifetime **im**aging (FLIM)-compatible small molecule, N(4-**a**mino**b**utyl)**p**henanthridiunium (ABP) with the brightness, spectral features, sensitivity to chloride, and selectivity versus other anions to serve as a useful probe of Cl_o_. ABP can be conjugated to dextran to ensure extracellular compartmentalization, and a second chloride-insensitive counter-label can be added for ratiometric imaging. We validate the utility of this novel sensor series in two sensor concentration-independent modes: FLIM or ratiometric intensity-based imaging.

## 1. Introduction

Chloride conductance through γ-aminobutyric acid (GABA) receptors (GABA_A_Rs) underlies the principal neuroinhibitory pathway in the brain. Changes in the relative concentrations of chloride across the neuronal membrane can impact the effectiveness of canonical neuroinhibition [1,2]. The direct, non-invasive measurement of chloride concentration (Cl^−^) in both the intracellular (Cl_i_) and extracellular (Cl_o_) compartments is of critical importance to study physiological processes relevant to several disorders, particularly epilepsy. The ratio of Cl_i_ to Cl_o_ is critically important to GABA_A_R functional neurological effects at the cellular and network levels. Cl_i_ can be measured using organic small molecules such as MEQ [3] or transgenic proteins such as Chlomeleon [4,5], SuperClomeleon, [6] or CloPhensor [7]. However, available transgenic proteins do not distribute to the extracellular space, and both transgenic proteins and small organic molecule probes are optimized for typically low Cl_i_ and lack the dynamic range to reliably report Cl_o_, which is typically an order of magnitude greater than Cl_i_. Verkman and colleagues developed several well-used small molecule fluorescent Cl_i_ sensors optimized for low chloride concentrations typical of the intracellular space, including SPQ [8,9], MQAE [10], and MEQ [3]. Importantly, this set of papers included structure–activity relationship information for several series of structures including those considered unsuitable for Cl_i_ measurement [10,11,12]. Undesirable properties in this low-chloride context were quite desirable when measuring higher concentrations of chloride typical of the extracellular space. These papers from the Verkman group provided us a solid structure–function relational foundation to develop a new fluorescent sensor optimized for Cl_o_ measurement.

Our initial efforts to measure Cl_o_ with commercially available fluorophores were subject to three primary limitations. First, the fluorophores demonstrated low sensitivity to changes in Cl_o_ at the relevant concentrations. The available chloride-sensitive fluorophores were optimized for low millimolar Cl^−^ typical of Cl_i_. Each was almost completely quenched at physiological Cl_o_. The dynamic range of these fluorophores is exhausted at concentrations greater than 20–40 mM, whereas Cl_o_ reported from ion-sensitive electrode studies is greater than 100 mM [13,14]. Second, the fluorescence emission lifetimes of existing probes at high Cl^−^ overlapped with short-lifetime autofluorescence in organotypic hippocampal slice cultures (HSCs). This issue was itself caused by two separate factors. The short wavelengths required for excitation of the available probes overlapped with the excitation wavelengths of intracellular autofluorescent sources. Additionally, the short lifetimes associated with near fully quenched chloride-sensitive fluorophores overlapped with the typical emission lifetimes of autofluorescent sources. Finally, available probes had inadequate brightness at the high end of the desired calibration range. Thus, even modest autofluorescence resulted in considerable contamination of the signal from available chloride-sensitive probes leading to questionable accuracy of Cl_o_ measurements. We synthesized several other fluorophores described by Verkman and colleagues [15] but did not find a compound that surmounted the difficulties in Cl_o_ measurement described above. We therefore sought to modify the compounds described by Verkman and colleagues to develop a suitable Cl_o_ reporter using the synthetic repertoire described therein. 

We describe the rational development of novel chloride-sensitive fluorophores optimized for concentration-independent **f**luorescence **l**ifetime **im**aging (FLIM) of Cl_o_. We present three related compounds: trimethyl-capped N-(n-aminoalkyl)phanthridinium, dextran-conjugated N-(n-aminoalkyl)phanthridinium, and a dual conjugated dextran with both chloride-sensitive N-(n-aminoalkyl)phanthridinium and a chloride-insensitive reference fluorophore. Dextran conjugation not only ensures extracellular localization of the fluorophore, but it also theoretically minimizes auto-quenching self-interactions that can occur at higher concentrations of fluorophore [11,16]. Conjugation to a large polymer also provides alternative utilization as a ratiometric reporter when a second spectrally distinct and chloride-insensitive fluorophore is conjugated to the same parent dextran. We provide initial experiments validating this dual-labeled dextran approach.

## 2. Methods

### 2.1. General

Chemicals were purchased from Sigma-Aldrich (St Louis, MO, USA) unless otherwise noted. Solutions were brought up in distilled water unless otherwise noted. Standard aCSF was brought up in 18 MΩ water and consisted of (in mM) NaCl 126; KCl 3.5; CaCl_2_ 2; MgCl_2_ 1.3; NaH_2_PO_4_ 1.2; Glucose 11; NaHCO_3_ 15. Low-chloride aCSF was identical, except the fact that sodium gluconate was substituted for NaCl, resulting in a final Cl^−^ concentration of 10 mM. Calibration solutions were prepared by mixing appropriate proportions of standard aCSF with low-chloride aCSF such that a chloride and gluconate sum total of 136 mM was maintained while Cl^−^ ranged from 10 to 136 mM. Colorimetric assays were read using a Wallac Victor-2 1420 spectrophotometer with a halogen continuous wave light source and spectral line filters at listed wavelength +/− 5 to 10 nm. Centrifugation steps were accomplished in a tabletop Eppendorf 5417R microcentrifuge. Reflux apparatus for synthesis consisted of a recirculating cooling bath (Thermo Fisher Scientific, Waltham MA, USA) filled with ethylene glycol cooling a 24/40 double-lumen coiled reflux tube (Ace Glassware, Vineland, NJ, USA) with a 500 mL round bottom flask (Owens-Corning, Corning NY, USA) heated with a heating mantle regulated by a timed power controller (Glas-Col #O406 and #104A PL312, respectively). 

### 2.2. Synthesis of Candidate Fluorescent Compounds

Synthesis techniques closely followed those of the Verkman group when synthesizing related compounds [11,15] and conjugating to dextran [11]. Briefly, alkyl chains were added to nitrogen heterocyclic organic backbones using either (1) sultones of a ring size corresponding to desired alkyl chain length when adding a sulfate group [15] or (2) an alkyl chain bearing a reactive bromide on one terminal carbon and on the other terminal carbon either a primary amine protected by a phthalimide group or else a trimethylated ammonium group [11]. In the case of alkylsulfate compounds such as NBSQ, sultone was mixed with isopropanol (1 part to 9 parts) in a 500 mL roundbottom flask with small Teflon-coated stir bar to which isoquinoline was added at a 2:1 molar ratio to sultone. For NBSQ synthesis specifically, we used 1 mL 1,4-sulfobutane to 9 mL isopropanol to which 0.635 g of isoquinoline was added and heated under N_2_ at 100–105 °C for 1 h [11,15]. Nitrogen was delivered via a 18G needle through a rubber septum covering flask neck. During this time, the reaction mixture changed from yellow–brown to bright yellow–green. The product was extracted in the aqueous phase from chloroform/water (4 parts to 1) in a separatory funnel. With a product volume of approximately 10 mL considered aqueous in relation to chloroform, we added 40 mL of chloroform and separated in a 125 mL separatory funnel. Chloroform was added to the resultant aqueous layer (4 parts to 1) and was again separated. The aqueous layer containing the product was then recrystallized from 95% ethanol twice. We note that changing the ring size of the sultone (1,n-sulfoalkyl) determines the length of the alkyl chain of the final product. 

In the case of alkylamine compounds, 10 g N-(n-bromoalkyl)trimethylammonium for quaternary amines or 10 g N-(n-bromoalkyl)phthalimide for primary amines was dissolved with an equimolar amount of phenanthridine in 30 mL acetonitrile (anhydrous) and refluxed for 20–24 h. Cooled reaction mixtures were filtered through Whatman-40 paper and rinsed with acetonitrile before recrystallization from 95% ethanol. The trimethylated moiety was produced in this single step while the phthalimide intermediate of the primary amine product required a deprotection step in the form of overnight reflux in aqueous acid (6N HCl) and subsequent filtration and recrystallization from 95% ethanol as after the first reaction step. Product purity was followed by thin-layer chromatography (TLC), and we found that a second recrystallization step was not necessary but may be performed without adversely affecting yield.

### 2.3. Synthesis of N(4-aminobutyl)phenantridinium (ABP)

Specifically for the compound we moved forward with, ABP, synthesis followed techniques described previously [11] for two closely related compounds, N(3-trimethylaminopropyl)phenantridinium and 6-methoxy-N(3-aminopropyl)quilolinium. N(4-***a***mino***b***utyl)***p***henantridinium (ABP) is a phenanthridine-based aminoalkyl moiety that is suitable for conjugation to dextran. Briefly, ABP was synthesized in a manner similar to N(3-trimethylaminopropyl)phenantridinium [11] by reflux of equimolar amounts of phenantridine and N(4-bromobutyl)phthalimide in acetonitrile overnight. A pale yellow–green precipitate was separated by filtration through Whatman-40 paper. From this intermediate phthalimide-protected compound, the phthalate group was removed by reflux in 6N HCl overnight and subsequent filtration, similar to procedures described for 6-methoxy-N(3-aminopropyl)quilolinium [11]. N(4-***a***mino***b***utyl)***p***henantridinium (ABP) was twice recrystallized from hot 95% ethanol/5% deionized water and was stored as its chloride salt in a sealed glass vial protected from light until conjugation to glucose polymer (i.e., dextran).

### 2.4. Conjugation of ABP to Dextran

Fluorophore conjugation to activated dextran was carried out according to a procedure lightly modified from Biwersi and colleagues [11]. Briefly, 800 mg of 10,000 Dalton (10 KDa) dextran (D9260, Millipore-Sigma, Danvers MA, USA) was dissolved in 10 mL of distilled water and activated by addition of cyanogen bromide at a 1:4 molar ratio to glucose units within the dextran. Adjustment of pH to 10.7 was accomplished with 10 M NaOH initially; then, 2 M NaOH until 10.7 pH was maintained for >10 min without further correction. ABP was added at a 1.1:4 molar ratio to glucose units within the dextran (10% molar excess over calculated binding sites). The pH was adjusted with 2 M NaOH to >9.3 and once maintained between 9.3 and 9.5 pH for >30 min without further correction; the mixture was allowed to stir overnight at 4 °C. The entire process was protected from light at all steps to as great a degree as possible while maintaining the requisite control over pH. The reaction was then quenched by addition of the Tris buffer (0.05 volumes of 1 M pH 8.0) and transferred to 3.5 KDa dialysis tubing (SpectraPor 132720, Thermo Fisher Scientific) and allowed dialysis against 50 volumes of distilled water changed twice daily for 4 changes, yielding 30–40 mL of 20–30 mg/mL of product. The resultant ABP-dextran was conjugated through its primary amine yielding a tertiary amine bound to one glucose unit within the polymer [11] at a final molar ratio of 4.4 ABP molecules conjugated to each 10 KDa dextran polymer. It should be noted that the considerable amount of chloride that may have been present in the ABP salt would have been dialyzed out during this process. ABP-dextran was confirmed resilient to both autoclaving and −20 °C freeze–thaw cycles and was thus aliquoted and stored under sterile conditions, protected from light. Once thawed, ABP-dextran could be kept at 4 °C for at least two weeks or if kept under sterile conditions for at least six weeks, again protected from light.

### 2.5. Dual-Labeled Dextran

In experiments in which dextran conjugation had a second step during which a chloride-insensitive red fluorophore was added, we used Alexa594 cadaverine (Thermo Fisher Scientific) and incubated ABP-dextran with this primary amine-bearing chloride-insensitive moiety for 30 min after overnight incubation with ABP, just before quenching the activated dextran (see above). With a greater linker length than ABP, we reasoned that Alexa594 cadaverine would be able to access potential binding sites from which ABP was sterically hindered. Full optimization of dual-labeled dextrans is planned but is beyond the scope of the present manuscript.

### 2.6. Spectrophotometry

Absorption readings were obtained using a Nanodrop One system (Thermo Fisher Scientific), while excitation and emission scans were obtained using a Spectramax M2 dual grating system (Molecular Devices, San Jose, CA, USA). Intensity scans relative to excitation wavelength obtained on either of our custom two-photon excitation (2PE) rigs were analyzed with ImageJ and plotted using Matlab (v2022a).

### 2.7. Fluorescence Lifetime Imaging (FLIM)

Time-correlated single-photon counting (TCSPC) FLIM measurements were obtained using one of two custom MaiTai Ti:Sapph laser-scanning two-photon microscopes with high-sensitivity photomultiplier tube (PMT) detectors. The first customized microscope was used for early *in vitro* experiments and is detailed in the *in vitro* imaging section below; the second was custom made for *in vivo* experiments and is detailed in the *in vivo* imaging section below. In either case, ABP-dextran was excited at 760 nm and emitted photons were subjected to a 445/58 bandpass filter (Chroma Technologies, Bellows Falls, VT, USA) prior to detection using high-sensitivity PMTs. Specific FLIM collection software differed between the two set-ups and is detailed in the relevant sections below, but in either case, each pixel had an associated histogram expressing photon number vs. photon travel time (the delay between excitation and detection) which was fitted to a single exponential curve to derive a time constant *k*,

(1) $${A_{t}=A_{o}e}^{-kt},$$

denoting the fluorescence lifetime of the fluorophore derived from each pixel. As implied by Equation (1), fluorescence lifetime is an intrinsic property of the fluorophore and wholly independent of fluorophore concentration. However, fluorescence lifetime may be shortened (i.e., quenched) by one or more moieties in a manner dependent upon the concentration of the quencher—*not the concentration of the fluorophore itself*. In the case of the fluorophore used in the present study (ABP), chloride is the sole biologically relevant quencher. The degree of lifetime shortening relative to the initial unquenched lifetime is linearly related to the concentration of the quencher through the Stern–Volmer relationship,

(2) $$\frac{\tau_{o}}{\tau_{i}}=1+\left[Q\right]k_{SV},$$

where *τ_o_*/*τ_i_* represents the unquenched lifetime divided by the shortened lifetime at a given quencher concentration ([*Q*]), necessarily yielding [17] a y-intercept of 1. The slope of the resultant plot, referred to as the Stern–Volmer constant (*k_SV_*), facilitates conversion of measured fluorescence lifetime to concentration of quencher. Notably, there is no term to describe fluorophore concentration in Equation (2) as the fluorophore concentration has no impact on measured lifetime. Only the concentration of the quencher, chloride, affects the fluorescence lifetime. Images are acquired over 1.5 min (*in vivo* and agarose gels) to 2.5 min (*in vitro* slice cultures). Each pixel is characterized using a histogram of photons populating time bins from which a single exponential time constant (fluorescence lifetime; the term *k* in Equation (1)) and intensity value (summed photons from all bins for that pixel) is derived. Of note, our optimization was performed with future experiments involving co-expression of certain intracellular fluorophores in mind, specifically Clomeleon [4,5] and Super Chlomeleon, [6] resulting in our use of 760 nm excitation instead of ABP-dextran peak at 770 nm. We found that 760 nm was the longest excitation wavelength at which we observed no measurable Chlomeleon or Super Chlomeleon excitation. Exciting ABP-dextran at 760 nm, only 10 nm off its peak excitation, was an acceptable compromise and we optimized ABP-dextran with this in mind. These efforts to minimize excitation of a fluorophore not directly relevant to ABP-dextran characterization are mentioned in Results in the interest of transparency, but in the interest of clarity, they are not belabored.

### 2.8. Calibration of ABP-Dextran

We initially calibrated ABP-dextran against chloride in a simple phosphate buffer (20 mM; pH 7.2) with varying amounts of NaCl (0–150 mM), such that solutions were essentially modified PBS. We also verified whether other biologically relevant anions were capable of quenching ABP using similar buffers with 20 mM phosphate and 0–150 mM anion or 4-(2-***h***ydroxy***e***thyl)-1-***p***iperazine***e***thane***s***ulfonic acid (HEPES) buffer (20 mM; pH 7.2) when varying phosphate concentration. For subsequent experiments performed in aCSF or *in vivo*, we calibrated ABP-dextran against aCSF with varying chloride concentrations. This was accomplished by preparing standard aCSF (in mM: NaCl 126; KCl 3.5; CaCl_2_ 2; MgCl_2_ 1.3; NaH_2_PO_4_ 1.2; Glucose 11; NaHCO_3_ 15) and a low-chloride aCSF in which NaCl was replaced with sodium gluconate, having demonstrated that gluconate had no appreciable effect on ABP-dextran quenching in preliminary experiments. We mixed appropriate amounts of this low-chloride aCSF with standard aCSF to achieve varying chloride concentrations (10–136 mM) in which the sum of sodium chloride and sodium gluconate was kept constant at 136 mM. FLIM calibrations were obtained for each batch of ABP-dextran on the same microscope and with the same range of laser powers used in experiments, though *k_SV_* did not appreciably change between ABP-dextran batches. Because calibrations performed in aCSF have a minimum Cl^−^ of 10 mM as described, the unquenched lifetime (*τ_o_*) used for Stern–Volmer calculations was approximated as the y-intercept (Cl^−^ = 0 mM) of a best-fit curve of the raw calibration data; empirically, a biexponential fit was used. All calculations were otherwise perfomed as described under the *Fluorescence Lifetime Imaging (FLIM)* section above. 

### 2.9. Animals

All animal protocols were approved by the Massachusetts General Hospital Institutional Animal Care and Use Committee. Wild type (C57bl/6J; Jackson Labs 000664), and Clomeleon mice (C57bl/6J background; Jackson Labs 013162) of either sex were used for this study. Mouse pups remained in the home cage with the dam under standard husbandry conditions until postnatal Day 6 to 8 (P6–8) when organotypic slice cultures were prepared or until a cortical window was surgically placed at P26–30.

### 2.10. In Vitro Organotypic Slice Cultures and Imaging

Organotypic hippocampal slice cultures were prepared either as glass-mounted [18] or membrane insert-mounted [19] cultures. Briefly, in either case, hippocampi were obtained from P6–8 mice and cut to 400 μm thick slices. These were then gently transferred to a 6-well dish containing either a membrane insert (Millipore) or a poly-L-lysine coated coverslip (Electron Microscopy Sciences), were fed twice weekly with 1 mL neurobasal-A media supplemented with 500 μM Glutamax, 2% B-27, and 0.03 mg/mL gentamycin (all from Invitrogen), and incubated at 35 °C in a 5% CO_2_ incubator. Cultures were imaged between DIV 7 and 21 in aCSF warmed to 33 °C and bubbled with 95%O_2_/5% CO_2_ perfused at a rate of approximately 100 mL/h unless otherwise noted. Slices were pretreated with 500 μg/mL ABP-dextran for 1–2 h prior to perfusing with aCSF containing 136 mM chloride and the same concentration of ABP-dextran. Slices were allowed equilibration in perfusate for 20–30 min before imaging, equaling at least 2 h of total exposure to ABP-dextran prior to initiation of imaging. Two-photon images were captured with a 20x water-immersion objective, NA 0.90, on a customized Olympus BX50WI microscope equipped with an 80 MHz Ti:Sapphire MaiTai laser (SpectraPhysics) driven with customized software for microscope operation and Becker and Hickel SPC800 FLIM hardware and software for data collection and initial processing in FLIM mode. Photons had to pass through a 445/58 bandpass emission filter before PMT detection (Hamamatsu C6438-01). Lifetime and intensity values for each pixel were generated using SPCImage software (v6.2, Becker & Hickel) before being exported for use in custom Matlab processing routines. 

### 2.11. In Vivo Murine Cortical Window Imaging

Using a modified method based on that of Che and colleagues [20], we placed cortical windows in young adult mice (P26–30) for acute (non-survival) imaging in accordance with Massachusetts General Hospital Institutional Animal Care and Use Committee policies and procedures (protocol 2018N000221). Briefly, mice of either sex were anaesthetized with isoflurane (maintained throughout experiment) and immobilized with ear bars and nosepiece. Scalp was cleared of hair using veterinary clippers and Nair^®^ hair removal cream, taking care to avoid the eyes and refreshing eye ointment after manipulations, and then sterilized with alternating cotton-tipped applicators soaked in either 70% isopropanol or 10% providone-iodine solution. In total, 0.1 mL of 0.4% lidocaine in sterile saline was injected under the area to be removed. After five minutes, a V-cut incision was made and a section of scalp was removed to expose a roughly 1 cm section of skull. Acrylic dental cement powder (Lang Dental, Wheeling, IL, USA) mixed with cyanoacrylate adhesive was used to fix the headbar to the exposed skull centered at 2 mm posterior and 3 mm lateral to Bregma, creating a 5 mm diameter working area. At this time a thick, peaking acrylic cement was desired and applied to the flat underside of the headbar around the margin of the opening for the cranial window and applied to the dry skull surface (see Che et al. [20] for dimensions and further details of the headbar). The central part of exposed skull that was the site of the cranial window was intentionally allowed visibe drying, though the tissue margin was periodically irrigated gently with aCSF-soaked gelatin foam applicator. Drying was allowed to occur so that (1) a firm attachment could form between the skull margin and the headbar and (2) the dura immediately underlying the skull at the surgical site could fuse to the overlying skull and be easily removed in the subsequent step. Tearing of the dura around the margin of the surgical site upon removal results in bleeding, but this is well-controlled with liberal irrigation using gelatin foam and is preferable to the use of forceps to remove the dura which typically results in more pronounced bleeding. A 2.5 mm round section of skull and underlying dura was removed using a dental drill (RamPower-45B, Ram Products, Dayton, NJ, USA) with a 0.5 mm bit (Fine Science Tools, Foster City, CA, USA) set to 30 rpm maximum. This is used to trace a circle halfway between the edge and the center of the 5 mm diameter headbar opening, resulting in a 2.5 mm diameter circle. Fine forceps were used to gently test the central area and to remove when mobilized. A 3 mm diameter No. 1 coverslip was placed over the exposed cortex. The coverslip had a 10 μL concentrated ABP-dextran/agarose mixture (a 1%*w*/*v* low gelation temperature agarose with 10 mg/mL ABP-dextran in aCSF) pipetted from 42 °C heat block immediately before inversion and placement. Liquid held to the underside of the glass, dispelling air as the coverslip was placed, but cooled rapidly. The coverslip was expeditiously placed over exposed cortex. Fine forceps were immediately used to adjust the coverslip laterally as needed and, finally, to gently press coverslip at opposite margins to meet the skull. Agarose containing ABP-dextran remained liquid for a brief time, and excess agarose was expelled from coverslip margins. Reposition of glass after this step was not attempted. Strips of Whatman-40 filter paper can be used to clear any large pieces of gel; small remnants were ignored. Cyanoacrylate was carefully applied to the margin of the coverslip, taking care not to allow any adhesive to run into the surface of the glass. Dental cement was prepared and applied over the dried cyanoacrylate, serving to both seal the edges of the window and to cover the remaining exposed organic tissue. One hour was allowed before image acquisition for ABP-dextran diffusion into cortex from overlying agarose (between cortex and coverslip); during this time, the warmed breadboard with immobilization apparatus and anesthetized mouse was transferred to the *in vivo* imaging microscope, a custom-made gantry-type two-photon microscope equipped with a MaiTai 80 MHz Ti:Sapphire laser (Spectraphysics) and driven with customized ScanImage software (v2021.0.0, MBF Bioscience, Williston, VT, USA). Photons were detected by a high-sensitivity PMT (Hamamatsu C5594-12) after passing through a 445/58 bandpass emission filter (Chroma Technologies) and digitized using custom ScanImage software. All other components were obtained from Thor Labs. The initial analysis of raw FLIM data generating fluorescence lifetime data for each pixel was accomplished using ImageJ plug-in FLIMJ. Subsequent analyses and plot generation were accomplished using custom routines in Matlab. Statistics were generated using relevant Matlab functions (Matlab v2022a, including statistics toolbox) and are reported in figure legends, as figure annotations, or both.

### 2.12. Image Processing in ImageJ and Matlab

Lifetime images are obtained either through Becker and Hickel (GmBH) proprietary software (SPCImage) or using the FLIMJ plug-in as part of ImageJ. Each concentration of Cl^−^ in the calibration curve has a normally distributed set of ABP-dextran lifetime values with a near-constant coefficient of variation (standard deviation divided by mean), except at particularly low Cl^−^ values where the coefficient of variation is greater (see Results). Fluorescence lifetime has a physical upper and lower bound over which its values are meaningful (lifetime in the absence of quencher vs. fully quenched fluorophore). Specifically for ABP-dextran, we find lifetime values to be linearly related to Cl^−^ from 0 mM to 150 mM through the Stern–Volmer relationship. We use these bounds for image processing as follows. Pixels are excluded (set to black) if ABP lifetimes are in excess of the mean lifetime in 0 mM chloride, or if lifetimes are shorter than twice the standard deviation less than the mean lifetime in 150 mM chloride (calculated from best-fit curve to raw calibration data and average coefficient of variation). Excluding all values outside this calibrated range represents an average exclusion of 0.5–1.5% of pixels obtained using Becker and Hickel FLIM (SPCImage) and 0.1–0.5% of pixels using the custom-built two-photon microscope described above.

### 2.13. Statistics

Statistical analysis was performed with Matlab 2022a, with access to statistical toolbox functions. Individual tests used are detailed in figure legends. Corresponding *p*-values are indicated within figures, in the associated figure legends or both.

### 2.14. Comaprative Brightness and k_SV_ Assay

Cl_o_ probe candidates are prepared in each of a set of calibration solutions containing chloride and gluconate totaling 136 mM in triplicate and fluorescence is read in a BioTek Synergy multifunction plate reader using bandpass filters, with excitation at 360/20 nm and emission at 440/50 nm. The Stern–Volmer constant (*k_SV_*) is calculated from fluorescence intensity data as described above from means of triplicate samples converted to a Stern–Volmer plot and fitted using linear regression, the slope of which yields *k_SV_*. Relative brightness is determined in 10 mM Cl^−^ as this is the minimum concentration used.

## 3. Results

### 3.1. Development of a Fluorescent Probe for Cl_o_

**Figure 1:**
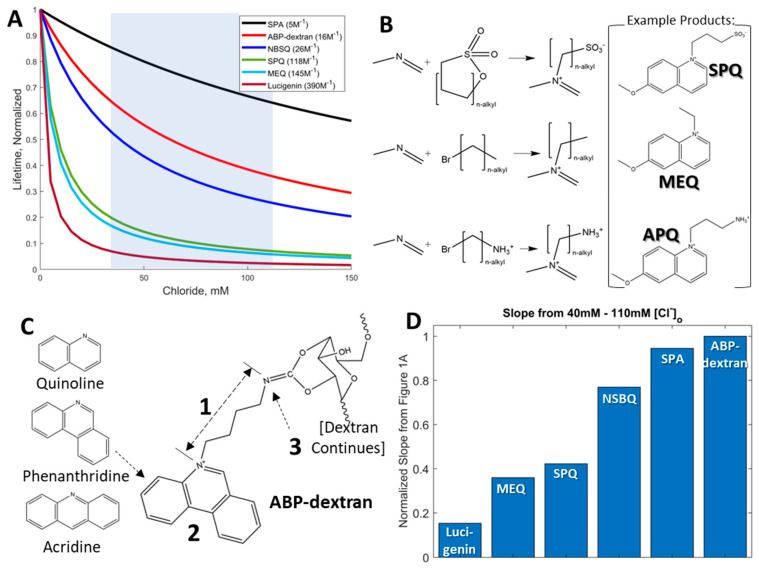
Limitations of existing chloride-sensitive fluorophores: Commercially available chloride-sensitive fluorophores were developed to measure intracellular concentrations of chloride (Cl_i_), which are typically an order of magnitude less than extracellular chloride concentrations (Cl_o_). (**A**) Sample data generated from published parameters of several chloride-sensitive probes and their published *k_SV_* values (see legend), each normalized for co-plotting, with the notable addition of our novel sensor ABP-dextran and empirical *k_SV_*. The most suitable Cl_o_ sensors have a large part of their total lifetime shortened, and emission quenched, as Cl_o_ increases from physiological Cl_i_ values to typical CSF values (i.e., 40–110 mM; shaded region). (**B**) Three ‘generations’ of heterocyclic addition chemistry applied to chloride measurement. The addition of sultones, bromated alkyl chains, and bromated alkylamines give rise to commercially available chloride sensors (SPQ and MEQ, respectively) as well as APQ, the most closely related Verkman series compound to ABP. Each example compound here has a (6-methoxy)quinoline base. (**C**) Several parameters were tuned in order to fashion a chloride sensor that met each of our stated criteria. The alkyl chain length was increased to increase *k_SV_*, but at the expense of overall brightness (1); a more complex heterocyclic structure was employed to red-shift excitation out of spectral overlap with autofluorescent species, taking care not to red-shift emission past the blue spectrum (2); we altered the trimethylated terminal nitrogen to allow conjugation to dextran and resultant extracellular sequestration (3). (**D**) Focusing on chloride concentrations greater than 40 mM up to typical CSF chloride of 110 mM (shaded region in (**A**), the linear slope of each compound was calculated and compared. NBSQ and SPA were improved by this metric but suffer from unacceptably short excitation wavelength and long emission wavelength, respectively.

**Figure 2:**
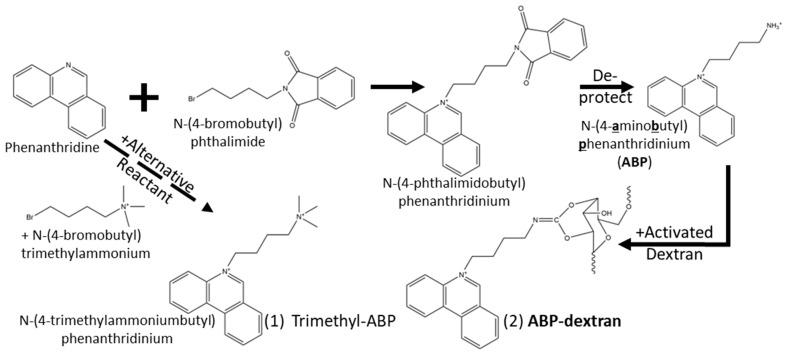
Synthesis of N-(4-aminobutyl)phenanthridinium (ABP) and Optional Conjugation to Dextran: Heating phenanthridine together with N-(4-bromoalkyl)phthalimide in anhydrous acetonitrile (ACN) under reflux conditions results in nucleophilic substitution of the bromide onto the heterocyclic nitrogen, yielding either N-(4-phthalimidobutyl)phenanthridinium intermediate or, alternatively, the derivative product N-(4-trimethylammoniumbutyl)phenanthridinium (Compound 1) if N-(4-bromoalkyl)trimethylammonium is reacted with phenanthridine instead. Conjugation to dextran is accomplished by first de-protecting the primary amine of the intermediate compound by reflux with aqueous acid (6N HCl), resulting in N-(4-aminobutyl)phenanthridinium (ABP). Activated dextran, having been pretreated with cyanogen bromide as described, is then incubated with ABP resulting in a fluorescent dextran labeled at several sites along the polymer (Compound 2).

Commercially available chloride-sensitive fluorophores have their origins in the Verkman group’s adaptation of Urbano’s heterocyclic chloride-sensitive compounds [8,21] into biologically compatible probes that were developed and optimized for low millimolar intracellular chloride [3,10,11,12,15,22] (Figure 1). These chloride-sensitive fluorescent molecules are quenched by halides, with different kinetic and photophysical properties. From the original sensors based on a derivatized (6-methoxy)quinoline base (SPQ [8,9,21] and MEQ [3]) to lucigenin [12], the largest and most sensitive Cl^−^ sensor incorporating paired acridine heterocyclics, these early examples share well-described structure-activity relationships. However, they are also optimized for measuring intracellular chloride concentration (Cl_i_), which is typically less than 10 mM and an order of magnitude less than expected extracellular chloride concentration (Cl_o_). These series of structures underlying the commercially available, collisionally quenched fluorescent chloride sensors have sensitivities to Cl^−^ that are well matched to Cl_i_ but not Cl_o_. A probe that is less sensitive to chloride overall retains more of its dynamic range at higher chloride concentrations and is thus more sensitive to Cl^−^ at higher chloride concentrations, making such a probe a better match for measurement of Cl_o_ (Figure 1A). Starting with probes rejected as Cl_i_ sensors for their perceived inadequate Cl^−^ sensitivity, we recognized these as potential Cl_o_ probes. While each of the known compounds had one or more challenges to circumvent, the wealth of structure–activity relationship information contained in a series of papers from the Verkman group [11,12,15] (Figure 1B) allowed us the use of well-defined chemistries and functional relationships to create a new probe that met our experimental needs. We utilized an asymmetrical heterotricyclic base structure to redshift the excitation wavelength into a range that did not excite endogenous autofluorescent compounds. Next, we tuned the length of the alkyl chain separating two nitrogens at either end to modify the chloride sensitivity of the probes. Then, we modified the distal nitrogen to a primary amine for conjugation to dextran to make N-(4-***a***mino***b***utyl)***p***henantridinium (ABP; Figure 1C). One way to measure how compatible candidate fluorescent probes are for Cl_o_ measurement is to compare the linearized slope of fluorescent lifetime plotted against Cl^−^ over the expected range of Cl^−^ one needs to measure (Figure 1A, shaded region). Cl_i_ is rarely recorded at greater than 40 mM, and Cl_o_ in the brain interstitial space is expected to be equal to that of CSF (110 mM); thus, we compared probes’ change in fluorescent lifetime over this range of Cl^−^ (i.e., fluorescence lifetime dynamic range from 40 to 110 mM Cl^−^; Figure 1D). Probes optimized for Cl_i_ measurement had poor dynamic range at these concentrations, being mostly quenched at Cl^−^ below 40 mM. N-4(sulfobutyl)isoquinolinium (NSBQ) appeared promising [15], but despite altering properties to make derivative compounds, the issue of an autofluorescent signal was persistent for this series due to the short excitation wavelength (710 nm using 2PE). Another promising candidate was N-sulfopropylacridinium (SPA) [12], but the expected dimness of the probe combined with its red-shifted emission wavelength that occupied the blue-green spectral space needed for intracellular chloride-sensitive fluorophores made SPA less useful for our purposes. ABP has appropriate red-shifting of excitation but not emission wavelength, a tuned alkyl chain length to alter sensitivity to Cl^−^ to the desired concentration range, and a primary amine to allow extracellular compartmentalization by conjugation to 10 kilodalton (10 KDa) dextran. Synthesis of N-(4-***a***mino***b***utyl)***p***henanthridinium (ABP) was informed by established methods [11] for synthesizing related compounds 6-methoxy-N-(3-***a***mino***p***ropyl)***q***uinolinium (APQ) and N-(***3***-***t***ri***m***ethyl***a***mmonium***p***ropyl)***p***henanthridinium (3TMAPP), modified for our purposes (Figure 2). Briefly, ABP was synthesized by equimolar reflux of phenanthridine with a phthalimide-protected primary amine-bearing compound (N-(4-bromobutyl)phthalimide) followed by deprotection and subsequent conjugation of the primary amine to dextran activated with cyanogen bromide (see Section 2). The related compounds with a trimethylated ammonium group that do not allow conjugation to dextran (e.g., N-(***4***-***t***ri***m***ethyl***a***mmonium***b***utyl)***p***henanthridinium; 4TMABP) are achieved in a single synthetic step (Figure 2; Compound 1 in lower left) and would be useful if proven to remain in the extracellular space. Notably, due to a gap in the catalog of our suppliers, we could not make the corresponding four-carbon-spaced quaternary version (4TMABP) for direct comparison with ABP-dextran. Comprehensive comparison of several pairs of different alkyl chain lengths is beyond the scope of this study.

### 3.2. Initial Characterization of ABP and ABP-Dextran

**Figure 3:**
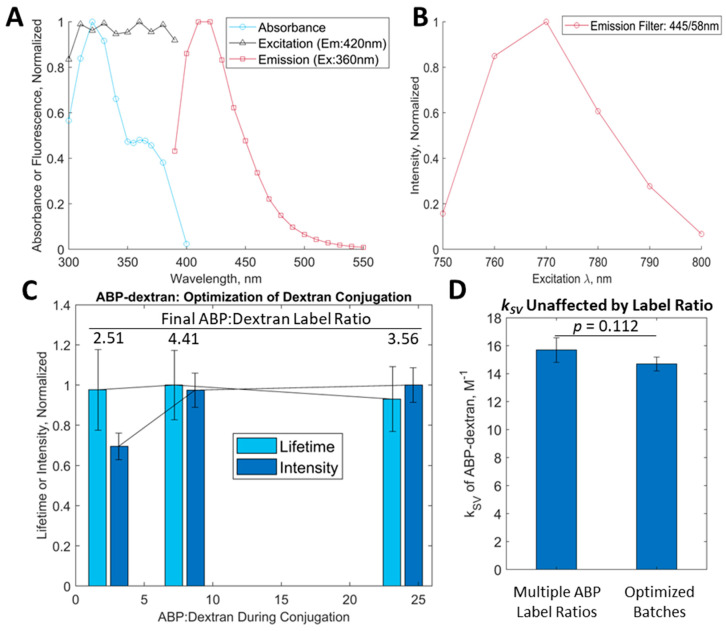
Characterization of novel chloride-sensitive probe ABP and its conjugation to dextran: ABP was synthesized as a primary amine amenable to dextran conjugation (see Figure 2). (**A**) Fundamental optical properties of ABP demonstrate a bimodal absorbance peak (blue line) with similar fluorescence excitation throughout (black line). Fluorescence emission data were gathered at 360 nm excitation and demonstrated a peak at approximately 420 nm. (**B**) Two-photon excitation (2PE) of ABP-dextran at given wavelengths through a 445/58 emission filter. We note that optical data generated from ABP conjugated to 10 KDa dextran (ABP-dextran) are shown; properties of unconjugated ABP were not appreciably different. (**C**) Characteristics of ABP-dextran as a function of molar ratio of primary amine ABP reagent to 10 KDa activated dextran. Pairs of bars are centered at molar ratios of 2.4, 8, and 24 ABP:dextran; calculated molar ratios achieved (‘final’) are annotated above pairs of bars. Intensity at lowest molar ratio of ABP added to dextran is significantly attenuated (2.4× vs. 8× *p* = 0.032; 2.4× vs. 24× *p* = 0.005), but greater molar ratios of ABP to dextran failed to significantly impact intensity readings (8× vs. 24× *p* = 0.220) consistent with all available conjugation sites being occupied. As expected, ABP-dextran lifetime did not change with increased ABP loading because fluorescence lifetime is not dependent on fluorophore concentration and Cl^−^ was held constant (*p*-value range: 0.5765 > *p* > 0.7538). (**D**) Calibrations of each ABP-dextran studied in (**C**) were carried out to obtain Stern–Volmer constants (*k_SV_*) for each, a measure of chloride sensitivity (see Methods). *k_SV_* did not change appreciably among dextrans with differing ABP label ratios (left bar; mean +/− SD: 15.7 +/− 0.8 M^−1^; *p* = 0.0) nor was *k_SV_* significantly different from later, fully optimized ABP-dextran batches (right bar; mean +/− SD: 14.7 +/− 0.5 M^−1^). (**C**,**D**): *mean +/− SD of lifetime or intensity data fit to normal distribution, i.e., mean +/− sigma(μ +/− σ), p-values reflect two-sample t-tests*.

We sought to excite our novel fluorophore at the longest wavelength possible to minimize any autofluorescent signal or tissue damage. ABP-dextran has a bimodal absorbance spectrum (Figure 3A, blue line) with a consistent fluorescent excitation profile (Figure 3A, black line). We chose to use excitation at 360 nm, the longest single-photon excitation wavelength that still achieves maximal excitation. Emission spectrum (Figure 3A, red line) is in the blue range with a maximum at 420 nm. Using two-photon excitation (2PE) microscopy, the ABP-dextran excitation maximum was determined to be 770 nm (Figure 3B), but moving forward, we chose to excite at 760 nm at the expense of 10–15% of emission intensity to minimize excitation of another fluorophore (SuperChlomeleon) we plan to use in future experiments (see Methods). Again referring to the work of the Verkman group [11], we further optimized the dextran activation and conjugation parameters to maximize the apparent number of conjugation sites and the amount of ABP to achieve an optimal labeling ratio of ABP:Dextran (Figure 3C). We found that while intensity initially increased with more ABP added to the reaction mixture, the fluorescence lifetime of ABP did not change appreciably (Figure 3C, dark blue and light blue bars, respectively). Once we exceeded an 8:1 molar ratio of ABP:Dextran in the conjugation reaction, the labeling ratio was maximized at approximately 4.4 moL ABP to 1 moL dextran (Figure 3C, annotation above each lifetime/intensity pair). There was no significant difference between the unquenched lifetimes of ABP-dextran prepared with different labeling ratios (Figure 3C, blue bars) nor was there a significant difference between the *k_SV_* calculated for these and the *k_SV_* calculated for the most recent batches of fully optimized ABP-dextran (Figure 3D).

### 3.3. Optimization of ABP-Dextran

**Figure 4:**
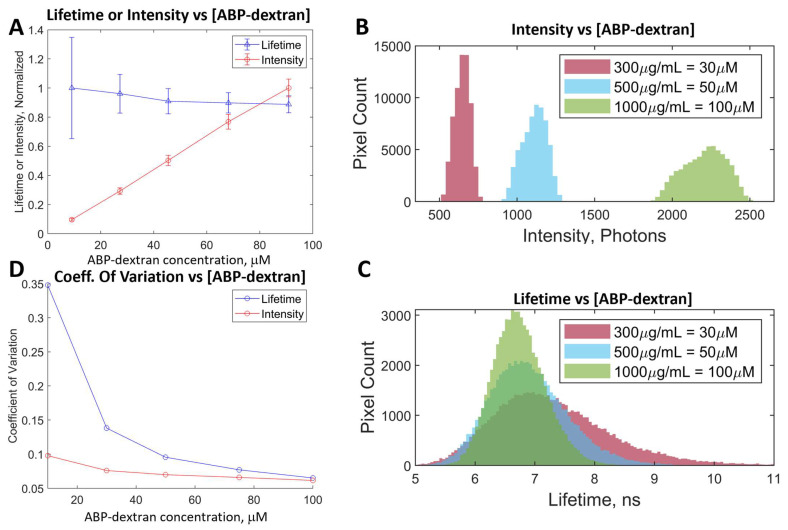
Optimization of dextran-conjugated ABP: Once ABP-dextran synthesis and conjugation were optimized, we next varied the concentration of this novel sensor to optimize experimental conditions. (**A**) Intensity, but not lifetime, increases linearly with ABP-dextran concentration from 100 μg/mL to 1000 μg/mL (10–100 μM). We note that as concentration of ABP-dextran increases, ABP lifetime variance decreases while mean lifetime changes only slightly. (**B**) Intensity histograms of three representative concentrations of ABP-dextran demonstrating that as ABP-dextran increases, both brightness and variance increase resulting in broader peaks of reduced amplitude. (**C**) Lifetime histograms comparing ABP-dextran concentrations corresponding to the data in (**B**) demonstrating that as ABP-dextran concentration increases, variance decreases. This creates narrower distributions of lifetimes with greater peak pixel counts as ABP-dextran concentration increases, even as intensity distribution broadens (**B**) over the same half log-step of ABP-dextran concentration shown in (**B**,**C**). This narrowing of lifetime distribution is asymmetrical, with longer lifetimes reduced more than shorter lifetimes as ABP-dextran concentration increases, resulting in the mean lifetime value drifting lower with increasing ABP-dextran (11.2% reduction over a 10-fold increase in concentration; (**A**), blue line) while the mode lifetime value changes < 1% from 50 μM to 100 μM ABP-dextran (blue vs. green) and only 5% for the half log-step data range shown. (**D**) Coefficient of variation decreases with increasing ABP-dextran concentration, most notably for lifetime values. (**A**): *mean +/− SD of lifetime or intensity data fit to normal distribution, i.e., mean +/− sigma (μ +/− σ)*; (**B**,**C**): *example histograms of raw data*; (**D**): *coefficient of variation (SD/mean) of the data displayed in* (**A**).

With a standardized synthetic process to make ABP-dextran, we next optimized experimental conditions with a FLIM-capable 2PE microscope using 760 nm excitation and a 445/58 bandpass emission filter (see Methods). Empirically, we found that around 200 μg/mL of ABP-dextran was necessary to obtain an adequate signal; thus, we tested ABP-dextran concentrations over one order of magnitude from 100 to 1000 μg/mL (i.e., 10–100 μM). We observed that while intensity increased linearly with ABP-dextran concentration, fluorophore lifetime was consistent but drifted to modestly lower mean values (Figure 4A, red and blue lines, respectively). When conjugated to dextran, we observed that ABP was mildly more resistant to self-quenching effects seen in non-conjugated fluorophores in this study observed at or near 300 μM. While more experiments need to be undertaken, we reasoned that ABP immobilization on dextran polymers reduces intermolecular interaction between ABP molecules, thus reducing self-quenching. Looking at histograms of raw data from three concentrations of ABP-dextran (Figure 4B,C), it can be seen that while intensity varies directly with histogram width (i.e., more signal results in greater variance; Figure 4B), lifetime varies inversely with histogram width (i.e., more signal results in less variance; Figure 4C). This likely is due to more photons detected in each pixel, yielding more data points from which fluorescent lifetime is calculated in each pixel (see Methods) and decreasing the variance of calculated lifetimes. This results in an asymmetric histogram tightening whereby the right shoulder (longer lifetimes) are lost preferentially over the left shoulder (shorter lifetimes) with minimal alteration of the mode (Figure 4C). This asymmetry explains the modest drift to shorter mean lifetimes with greater ABP-dextran concentration observed in Figure 4A (blue line). To quantify this variance, we plotted the coefficient of variation (standard deviation divided by mean) against ABP-dextran concentration (Figure 4D) and found that both lifetime and intensity measurements feature a decreasing coefficient of variation and appear to approach a shared asymptote (blue and red lines, respectively). This decrease in coefficient of variance with increasing ABP-dextran concentration is more pronounced in the case of fluorescence lifetime than intensity. We found that as long as ABP-dextran concentration is at least 500 μg/mL (50 μM), the fluorescence lifetime coefficient of variation remains acceptably, if arbitrarily, low (<0.1, arbitrary units). In all future experiments, we used ABP-dextran at 500 μg/mL unless otherwise noted, and we displayed calibration data as mean +/− standard deviation (SD) of a normal distribution fit of the data (see Methods).

### 3.4. ABP-Dextran Performance as a Cl_o_ Probe

**Figure 5:**
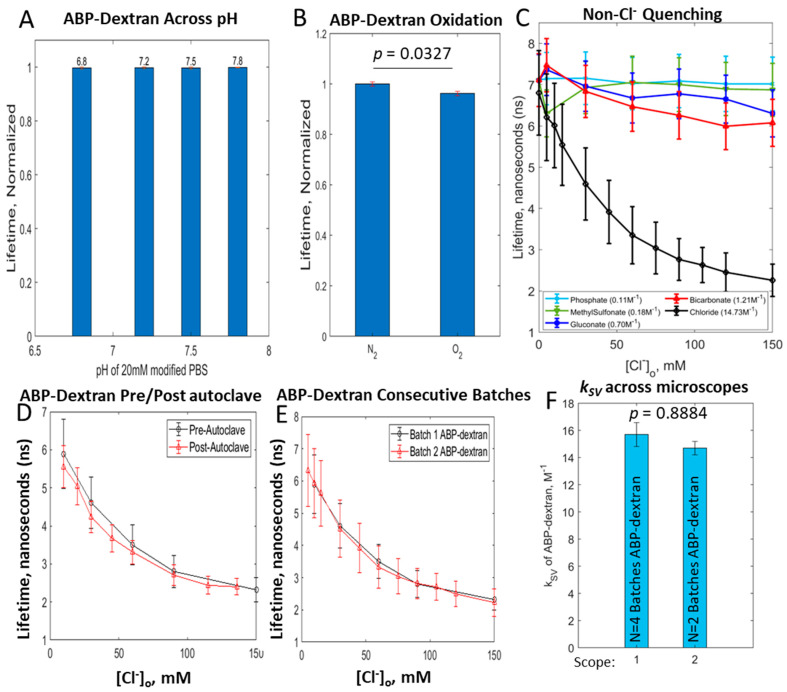
Reproducibility and performance of ABP-dextran. Having developed a suitable Cl_o_ probe, we next confirmed that our optimization was adequate to move on to *in vitro* and *in vivo* measurement of Cl_o_. (**A**) pH independence of ABP-dextran over the physiological concentration range (6.8–7.8 pH). (**B**) Bubbling with 95% O_2_/5% CO_2_ causes a statistically significant reduction in lifetime of less than 4% of unquenched signal. This stable level of quenching is experimentally acceptable as long as calibration solutions are also bubbled with 95% O_2_/5% CO_2_. (**C**) Absence of ABP-dextran induced quenching in the presence of other biologically relevant anions: phosphate, methyl sulfonate, bicarbonate and gluconate. (**D**) ABP-dextran performs similarly before and after autoclave, allowing long-term *in vivo* experiments. (**E**) Optimized synthetic and experimental procedures produce reproducible results batch to batch; consecutive batches of ABP-dextran shown. (**F**) Though separate FLIM-capable microscopes report differences in *k_SV_* due to hardware-associated discrepancies, when raw values of each rig are normalized there is no significant difference between the calculated *k_SV_* values, i.e., there is no difference in Cl^−^ sensitivity. (**A**): *mean +/− SD, no statistically significant differences*; (**B**): *mean +/− SD, unpaired t-test p = 0.0327*; (**C**–**E**): *mean +/− SD of normal fit to data at each [Cl^−^]*; (**F**): *mean +/− SD, unpaired t-test p = 0.888*4.

We next sought to functionally characterize ABP-dextran as a Cl^−^ reporter under experimental conditions anticipated in both *in vitro* and *in vivo* settings. This meant ruling out other potential sources of signal instability including batch-to-batch variation, survival after autoclave sterilization, and variation due to pH differences, oxidation, and other biologically relevant anions. We first determined that ABP-dextran was not sensitive to pH over the physiological range (6.8–7.8 pH; Figure 5A). Next, we compared ABP-dextran solutions bubbled with nitrogen or oxygen to test for oxidative quenching (Figure 5B). While we found an oxygen-dependent quenching effect of statistical significance, this modest effect of less than 4% was considered an acceptably small deviation given that calibration solutions used are similarly oxygenated. Next, we tested other biologically relevant anions for ability to quench ABP-dextran. While we already determined that sodium gluconate was an acceptable inactive substitute for sodium chloride when preparing calibration solutions of equivalent ionic strength, we formalized this observation and tested several other biologically relevant anions including phosphate, organic sulfate (methylsulfonate), and bicarbonate, each with measured *k_SV_* ≤ 1.2 M^−1^ (Figure 5C). These alternative anions possessed *k_SV_* ranging from 0.11 M^−1^ to 1.21 M^−1^ versus ABP-dextran *k_SV_* for chloride that was found to be 14.7 +/− 0.5 M^−1^ (mean +/− standard deviation). The inert nature of these biologically relevant anions in these experiments suggests that ABP-dextran can be used to measure chloride in a range of clinical samples. We verified that our novel modified dextran is just as versatile and stable as commercially available fluorescently labeled dextrans by subjecting it to freeze–thaw and autoclave cycles (Figure 5D) without affecting its characteristics as a chloride sensor. Finally, having optimized the synthesis of the ABP primary amine, the dextran activation and conjugation steps, and the experimental conditions on two microscopes with different FLIM hardware and software, we verified that ABP-dextran performed consistently (Figure 5E,F). ABP-dextran FLIM characteristics of consecutive synthetic batches were nearly identical, so that data almost overlay (Figure 5E). While microscope to microscope differences are expected, particularly with a technique that relies on measuring photon flight time, the differences in *k_SV_* calculated for each of six total batches of ABP-dextran was not significantly different between microscopes (Figure 5F). Our future experiments necessitate that this dextran-conjugated fluorophore is used in conjunction with intracellular fluorophores that occupy the typical green and red emission spectra. For this reason, we focused on ABP-dextran optimized for future co-labeled studies. While we left full characterization of trimethylated small-molecule variants N-(3-trimethylammoniumpropyl) phenanthridinium (3TMAPP) and N-(5-trimethylammoniumpentyl) phenanthridinium (5TMAPP) for future studies, we report their basic characteristics in Table 1. 

### 3.5. Performance In Vitro and In Vivo Mouse Tissue

**Figure 6:**
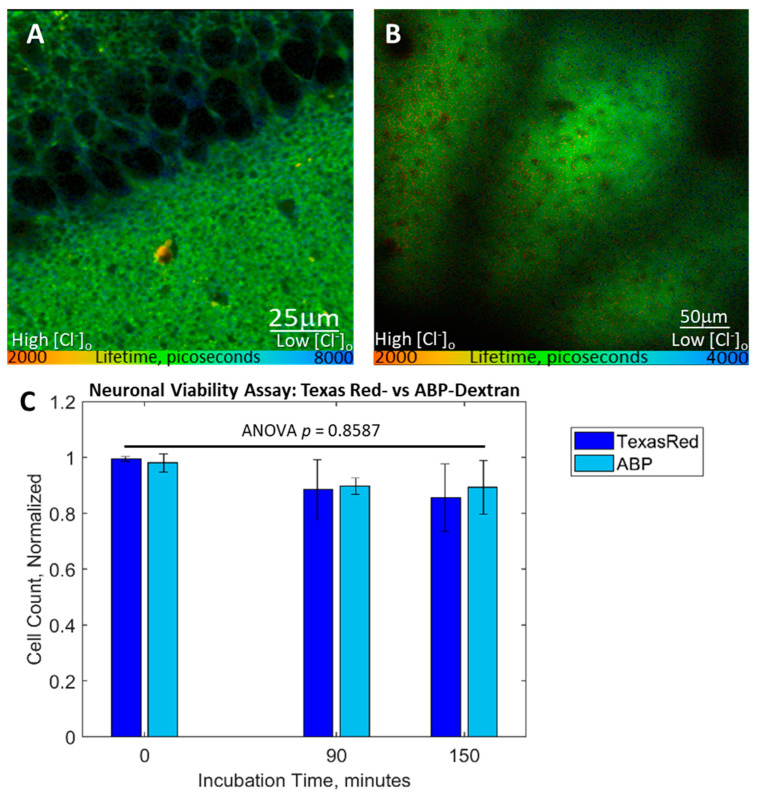
Use of ABP-dextran *in vitro* and *in vivo*. FLIM images from two separate microscopes are composites of intensity and lifetime components, where color encodes fluorescence lifetime. (**A**) Murine hippocampal organotypic slice culture (DIV8) perfused with 500 μg/mL (50 μM) ABP-dextran in aCSF, CA1 and stratum radiatum regions shown (top left versus bottom right, respectively). This image was taken with an older rig equipped with Becker and Hickel (B&H, GmbH) FLIM hardware and software (SPCImage, v6.2). (**B**) Murine *in vivo* image taken in a second, specially equipped custom rig with custom FLIM hardware and Vidrio (v2021.0.0) acquisition and FLIMJ analysis software. Layer 2/3 neocortex is shown as viewed through a cortical window placed at P27, at a depth of 178 μm from the overlying cortical surface. Large shadows are the result of blood vessels superficial to the field of view while small silhouettes are somatic shadows, each visible because of ABP-dextran exclusion from intraluminal and intracellular space, respectively. (**C**) Fluorescence quenching cytotoxicity assay performed in DIV12 HSCs expressing transgenic Chlomeleon fluorescent protein. Neuronal populations in each slice were followed for 2.5 h in culture media supplemented with 50 μM ABP-dextran (light blue bars) or Texas Red-dextran control (dark blue bars; both were 10 KDa dextran). HSCs had a non-zero rate of neuronal death as seen in (**C**), but ABP-dextran did not accelerate this rate and demonstrated no difference versus control. (**C**), *Each of three independent experiments for each condition were normalized to initial fluorescent CA1 neuron count; one-way ANOVA of normalized data, p = 0.8587 (ns)*.

ABP-dextran performed well both *in vitro* and *in vivo* in our mouse model system. Hippocampal slice cultures (HSCs) of wild-type mice were imaged at DIV7-14 after pretreatment with 500 μg/mL (50 μM) ABP-dextran in aCSF containing 136 mM Cl^−^ (see Methods). The representative image shown in Figure 6A details the CA3 pyramidal cell layer and associated stratum radiatum to the lower right, demonstrating silhouettes caused by ABP-dextran exclusion from neuronal cell bodies in stratum pyramidale and larger neurites and small vessels in stratum radiatum (Figure 6A, upper left and lower right, respectively). HSC was imaged 45 μm below the upper surface of the slice. The upper surface of the slice was determined from the change in fluorescence intensity occurring at the transition from aCSF to brain tissue. This transition was determined at the center of the field of view to account for the natural dome-shaped curvature that HSCs obtain over time. *In vivo* images obtained through a cortical window implanted in P26–P30 wild-type mice were obtained with a custom 2PE microscope built specifically for *in vivo* experiments (see Methods). ABP-dextran was delivered *in vivo* by mixing into agar at a 10 mg/mL final concentration. The agar was placed between the exposed cortex and overlying glass coverslip at the time of cortical window placement, allowing ABP-dextran passive diffusion into the cortex. Based on empirical comparison of ABP-dextran brightness *in vivo* with *in vitro* experiments at consistent experimental settings, final concentration of ABP-dextran in the mouse cortex was estimated to be between 400 and 600 μg/mL (40–60 μM). ABP-dextran was excluded from vessels and cell bodies *in vivo* (Figure 6B). We next sought to formally test ABP-dextran for cellular toxicity in a manner compatible with the HSCs we utilized throughout our experiments. Fluorescence quenching has been utilized in HSCs as a sensitive method to identify dying neurons early in the process, offering a stringent and validated test of toxicity [23,24]. ABP-dextran or commercially available Texas Red dextran was added to HSCs prepared from mice expressing Thy-1 driven chloride-sensitive intracellular reporter Chlomeleon [4,5] and followed for 150 min. Compared to control fluorescent dextran, ABP-dextran demonstrated no increase in toxicity (Figure 6C). While we are eager to utilize Chlomeleon and Super Chlomeleon-expressing mice in future experiments, wild-type animals were used for all other experiments in this manuscript so as not to introduce another fluorophore in these proof-of-concept experiments. However, the fact that ABP-dextran emission occupies the typical blue channel spectrum leaves the majority of spectral space, from green to infrared, open for co-labeling. We took advantage of this fact to explore conversion of our FLIM-optimized probe to a ratiometric probe that does not require measurement of fluorescence lifetime but only dual-channel fluorescence, a more convenient methodology for most laboratories.

### 3.6. Exploring a Ratiometric Cl_o_ Probe

**Figure 7:**
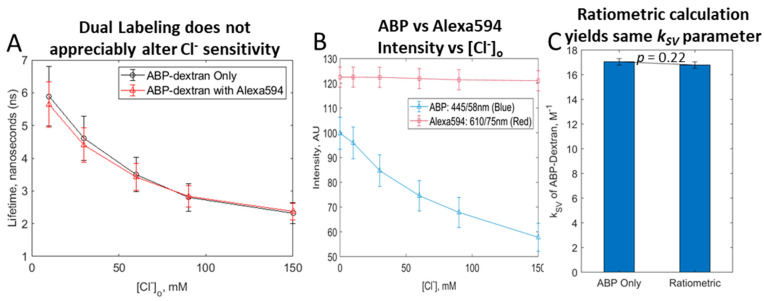
Dual-labeled ABP/Alexa594-dextran performs. (**A**) Dual labeling ABP-dextran with chloride-insensitive fluorophore Alexa-594 has no appreciable effect on chloride-induced ABP quenching. (**B**) ABP intensity is quenched with increasing chloride concentration but chloride-insensitive Alexa594 is not, as expected. The ratio of ABP intensity to Alexa594 intensity at each chloride concentration measured is used to calculate *k_SV_* as a ratiometric measure of Cl^−^. (**C**) Chloride-insensitive fluorophore Alexa-594 can be used to provide a ratiometric measurement, making intensity measurements alone sufficient to provide a Cl_o_ measurement independent of fluorophore concentration. (**A**,**B**) *mean +/− SD of normal fit to data at each [Cl^−^]*; (**C**) *mean +/− SD, unpaired t-test p = 0.2184*.

Dextran conjugation afforded us the opportunity to additionally label activated dextran with chloride-insensitive fluorophore to allow ratiometric Cl_o_ determination. Fluorescence lifetime imaging is already independent from probe concentration, but it requires investment in specialized hardware and typically demands long acquisition times. A ratiometric dual-labeled dextran could also provide spatial information independent of Cl^−^, which may allow better definition of physical features such as vessel or cell borders. Efforts have been made previously to construct such a ratiometric Cl^−^ sensor, but either these attempts were unsuccessful [11] or incompletely chloride-insensitive probes were used with minimal spectral separation from the chloride-sensitive structure [25]. At any rate, these previous attempts again focused on measuring low values of Cl_i_ and were subject to autoquenching effects. ABP-dextran was optimized for Cl_o_ and was resistant to autoquenching (see above). We first tested whether adding a chloride-insensitive label altered ABP-dextran chloride responsiveness. Splitting a batch of ABP-dextran into one half that was prepared as described and the other half that was incubated with Alexa594 just before quenching of dextran activated sites (see Methods), we were able to directly compare ABP-only labeled dextran to dual labeled dextran. Comparing these, we found that the presence of the chloride-insensitive red fluorescent label had no effect on ABP lifetime response to chloride (Figure 7A). The key advantage to using the dual label is the ability to rely on the fluorescence intensity ratio rather than the fluorescence lifetime to calculate *k_SV_* and thus chloride concentration. As expected, while ABP intensity decreased with increasing chloride concentration, the intensity of the Alexa594 signal remained unchanged (Figure 7B). The ratio of the ABP signal to the Alexa594 signal (blue:red ratio) was calculated and plotted against chloride concentration instead of lifetime (the Stern–Volmer plot), allowing the calculation of *k_SV_* obtained from this ratiometric treatment. The ratiometrically obtained *k_SV_* was not significantly different from the *k_SV_* obtained using direct fluorescence lifetime measurements (Figure 7C), confirming the accuracy of this alternative approach.

## 4. Discussion

Previous extracellular Cl measurements typically relied on chloride-selective electrodes with tip diameters of 3–5 microns [13]. Insertion of such instruments causes a stab-type wound that is likely to disrupt local extracellular matrix in intact tissue and is inherently invasive. The fluorescent chloride-sensitive probe described here enables the first truly non-invasive measurements of Cl_o_ *in vivo*. The key features of ABP as a useful probe of Cl_o_ included minimization of the autofluorescence artifact by virtue of sufficient fluorophore brightness (Figure 4B), sufficiently long minimum lifetime relative to autofluorescent lifetime, and excitation and emission spectra relative to autofluorescence (Figure 3); appropriate sensitivity to chloride concentrations found in the extracellular space (Figure 1); selective quenching of ABP by chloride vs. other anions present in the extracellular space (Figure 5C); excitation at sufficiently long wavelengths so as to avoid phototoxicity (Figure 3); and absence of toxicity (Figure 6).

While FLIM measurements have low time resolution, conjugation of chloride sensitive and insensitive fluorophores enables more rapid chloride measurements based on ratiometric intensity data. Ratiometric data facilitate physiological experiments characterizing the stability and plasticity of the extracellular chloride concentration. The ability to perfuse ABP over the cortex makes possible repeated measures of Cl_o_ over long intervals of time in vivo. Such time-resolved measures are critical to determine the long-term stability of the surprisingly heterogenous Cl_o_ values observed using ABP-dextran. Cl_o_ is an important determinant of GABA_A_ receptor-mediated signaling [26]. Local exclusion of Cl_o_ by sulfated moieties of the extracellular matrix [27] can then provide a novel mechanism for long-term memory. ABP makes it possible to test these intriguing hypotheses.

Our preliminary data suggest that the actual Cl^−^ in perineuronal space is lower than CSF Cl^−^, and we hypothesize that other less mobile anions such as chondroitin sulfate make up the remaining charge density [28]. Chondroitin sulfate is known to be abundant in the brain extracellular extracellular matrix (ECM) [29], and the immobile anionic charge concentration present in the perineuronal space can be as high as that of CSF [30]. Our data reveal that when measured non-invasively and without damage to the living tissue being measured, extracellular chloride concentration is less than CSF concentration *in vivo*, and less than aCSF perfusate *in vitro*. This unexpectedly low value of Cl_o_ creates the possibility of increases in Cl_o_ after brain injury. Removal of immobile anionic charges in the ECM as a consequence of matrix metalloproteinase activation as described for several injurious conditions [31,32,33,34,35,36] could result in Cl_o_ increasing to plasma and CSF concentrations [2]. Equilibrative neuronal membrane cation–chloride cotransporters can then increase Cl_i_, as has been demonstrated in several of these conditions [2], resulting in cytotoxic edema. Our novel Cl^−^ reporter allows the direct measurement of Cl_o_ and enables this hypothesis to be tested in model systems.

Our design of a novel fluorophore based on disfavored molecules from earlier Cl_i_ probe series is the result of both the rational design described in this paper and responses to several observations and potential critiques. For example, we were initially motivated to use a larger heterocyclic backbone to increase spectral separation of our probe from autofluoresence in hippocampal slice cultures. The autofluorescent signal was not a major issue *in vivo*, however, smaller probes such as isoquinoline-based moieties may still be of utility in this context. Likewise, although conjugation to dextran has additional advantages beyond exclusion from intracellular and intraluminal spaces, the trimethylammonium derivative of ABP (TMABP) may be more convenient for future research as its two fixed positive charges are likely to prevent it crossing intact cellular membranes. We initially chose to conjugate ABP to dextran out of an abundance of caution that longer than expected lifetime readings could be due to leakage of fluorophore into the low-chloride intracellular space. 

While we made every effort to optimize a Cl_o_ probe of maximal utility and generalizability across techniques and experimental approaches, we acknowledge that more parameter space remains to be explored. The complex aCSF buffers used herein indicate that ABP-dextran should be compatible with patient CSF and other clinical sample types such as plasma, urine, or sweat. However, this remains a future direction as we did not specifically test compatibility with these alternative sample types. In addition, we recognize that probes allowing selective measurement of extracellular sulfate can provide a useful compliment to ABP-dextran measurements of Cl_o_.

## 5. Conclusions

ABP-dextran, a novel chloride-sensitive fluorescent probe restricted to extracellular space, allowed the first truly non-invasive measurements of Cl_o_ in living systems. While we maintain our focus on ABP-dextran, related quaternary amine compounds 3TMAPP and 5TMAPP are likely membrane impermeant owing to multiple resident charges. Extracellular compartmentalization must first be verified, but these unconjugated small molecule variants of ABP-dextran may be preferable for Cl_o_ measurement in some systems. Each of these three probes are sufficiently bright, sufficiently sensitive and specific to chloride. Each allows reliable measurement of extracellular chloride due to relatively low Stern–Volmer constants (*k_SV_*) that allow the stretching of their dynamic range to higher chloride concentrations relevant to the extracellular space. Finally, each of these three probes offers sufficient spectral separation from common autofluorescent sources. We demonstrate these advantageous properties in mouse hippocampal slice cultures (HSCs) and *in vivo* in mouse using cortical windows, all in the absence of ABP-dextran toxicity.

## Figures and Tables

**Table 1:** Comparison of novel Cl_o_ probes and MEQ.

	Brightness ^a^	QY	*k_SV_*
MEQ	1.00	0.70 ^b^	145 ^b^
3TMAPP	0.29	0.74	41
5TMAPP	0.27	0.84	32
1° ABP	0.37	0.75	34
ABP-dextran ^c^	0.24	0.54	19

All fluorophores studied at 200 μM in modified aCSF (see Methods). MEQ, 6-methoxy(N-ethylquinolinium); 3TMAPP, N-(3-trimethylammoniumpropyl)phenanthridinium; 5TMAPP, N-(5-trimethylammoniumpentyl)phenanthridinium; 1° ABP, N-(4-aminobutyl)phenanthridinium; ABP-dextran, N-(4-aminobutyl)phenthridinium conjugated to 10 KDa dextran. ^a^: Brightness determined relative to MEQ, all at 200 μM. ^b^: MEQ characteristics as reported in Bisweri et al. [3]. ^c^: Due to ABP-dextran empirical label ratio of 4.4, 45.5 μM ABP-dextran was used to achieve 200 μM fluorophore concentration.

## Data Availability

Data is available upon request.
