# Correction: Normoyle et al. Synthesis and Characterization of a Novel Concentration-Independent Fluorescent Chloride Indicator, ABP-Dextran, Optimized for Extracellular Chloride Measurement. *Biomolecules* 2024, *14*, 77

**DOI:** 10.3390/biom16040515

**Published:** 2026-03-31

**Authors:** Kieran P. Normoyle, Kyle P. Lillis, Kevin J. Staley

**Affiliations:** 1 Massachusetts General Hospital, Department of Neurology, 55 Fruit Street, Boston, MA 02114, USA; kieran.normoyle@mgh.harvard.edu (K.P.N.); klillis@mgh.harvard.edu (K.P.L.); 2 Harvard Medical School, Department of Neurology, 77 Louis Pasteur Avenue, Boston, MA 02115, USA

In the original publication [1], there were several formatting errors caused through the reversion of subscript and character types. The most serious of these was a recurring character reversion resulting in several instances of Greek letters displayed as Latin characters throughout the publication. In some instances, the Greek letter μ (mu) was displayed as the Latin letter “m”, while in others it appeared as the Latin letter “u”. This resulted in concentrations misreported in various forms such as “mM”, “mg/mL” “uM”, or “um” when they should have read “μM”, “μg/mL,” or “μm”, with some errors reflecting concentrations one thousand-fold greater than intended. Other less impactful Greek letter errors also occurred, e.g., “g-aminobutyric acid” instead of “γ-aminobutyric acid.” Less important spelling and typographical errors, including subscript and italicization use, punctuation, and minor grammatical adjustments, have also been made consistent in this correction. Importantly, the concentration of the ABP-Dextran extracellular chloride sensor used was in the range of 10–100 μM (100–1000 μg/mL), not 10–100 mM (100–1000 mg/mL).

Additionally, in Figure 4, the x-axis labels of panels A and D displayed ‘m’ instead of ‘μ’ in the concentration units (μM). The same character encoding error appeared in the legends of Figures 4 and 6, where concentration units were displayed with ‘m’ rather than ‘μ’. The corrected Figure 4 appears below.

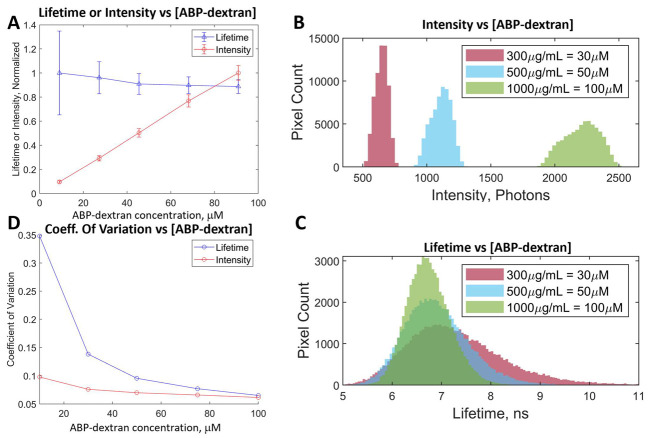



Furthermore, subscript formatting was not properly maintained in chemical notations, affecting the display of intracellular and extracellular chloride concentrations. The notation “Cl_i_” and “Cl_o_” appeared without proper subscripting in multiple instances throughout the manuscript.

The italicization of Latin phrases was also inconsistent, with in vitro and in vivo sometimes appearing in regular type instead of italics.

Additional corrections include: the reagent name ‘Alexa594’ corrected to ‘Alexa594 cadaverine’ in Section 2.5; the phrase ‘modest effect’ expanded to ‘modest effect of less than 4%’ and the symbol ‘kSV < 1.2 M^−1^’ corrected to ‘kSV ≤ 1.2 M^−1^’ in Section 3.4; ‘incompletely insensitive’ corrected to ‘incompletely chloride-insensitive’ in Section 3.6; and the abbreviation ECM introduced at its first use in Section 4.

Due to the pervasive nature of these formatting errors throughout the manuscript, corrections have been made to the following sections:

1. Abstract;
2. Introduction (multiple paragraphs);
3. Section 2.5: Fluorescence Lifetime Imaging (FLIM);
4. Section 2.7: Calibration of ABP-Dextran;
5. Section 2.9: Animals;
6. Section 2.10: *In Vitro* Organotypic Slice Cultures and Imaging;
7. Section 2.11: *In Vivo* Murine Cortical Window Imaging;
8. Section 2.14: Comparative Brightness and *k_SV_* Assay;
9. Section 3.1: Development of a Fluorescent Probe for Cl_o_;
10. Section 3.3: Optimization of ABP-Dextran;
11. Section 3.4: ABP-Dextran Performance as a Cl_o_ Probe;
12. Section 3.6: Exploring a Ratiometric Cl_o_ Probe;
13. Section 4: Discussion (multiple paragraphs);
14. Section 5: Conclusions;
15. All figure legends (Figure 1, Figure 3, Figure 4, Figure 5 and Figure 6, and 7);
16. Table 1 legend.

Furthermore, the name ‘Thomas Shiu’ in the Acknowledgments has been corrected to ‘Fu Hung Shiu’ to reflect the author's preferred professional name.

The authors state that the scientific conclusions are unaffected. All corrections were approved by the Academic Editor. The original publication has also been updated.

## References

[1] Normoyle K.P., Lillis K.P., Staley K.J. (2024). Synthesis and Characterization of a Novel Concentration-Independent Fluorescent Chloride Indicator, ABP-Dextran, Optimized for Extracellular Chloride Measurement. Biomolecules.

